# Anomaly detection in images with shared autoencoders

**DOI:** 10.3389/fnbot.2022.1046867

**Published:** 2023-01-04

**Authors:** Haoyang Jia, Wenfen Liu

**Affiliations:** ^1^Guangxi Key Laboratory of Cryptography and Information Security, Guilin University of Electronic Technology, Guilin, Guangxi, China; ^2^School of Computer Science and Information Security, Guilin University of Electronic Technology, Guilin, Guangxi, China

**Keywords:** anomaly detection, autoencoder (AE), unsupervised learning, adversarial network, image identification

## Abstract

Anomaly detection is a classical problem in computer vision, namely the determination of the normal from the abnormal when datasets are highly biased toward one class (normal) due to the insufficient sample size of the other class (abnormal). We introduce a novel model that utilizes two decoders to share two encoders, respectively, forming two sets of network structures of encoder-decoder-encoder called EDE, which are used to map image distributions to predefined latent distributions and vice versa. In addition, we propose an innovative two-stage training mode. The first stage is roughly the same as the traditional autoencoder (AE) training, using the reconstruction loss of images and latent vectors for training. The second stage uses the idea of generative confrontation to send one of the two groups of reconstructed vectors into another EDE structure to generate fake images and latent vectors. This EDE structure needs to achieve two goals to distinguish the source of the data: the first is to maximize the difference between the fake image and the real image; the second is to maximize the difference between the fake latent vector and the reconstructed vector. Another EDE structure has the opposite goal. This network structure combined with special training methods not only well avoids the shortcomings of generative adversarial networks (GANs) and AEs, but also achieves state-of-the-art performance evaluated on several publicly available image datasets.

## 1. Introduction

Anomaly detection is an important data mining method, which plays a very important role and significance in understanding data mining data. As early as Hawkins (1980) gave an accurate definition of outliers, that is: “Outlier data is so obviously different from most observed data that it is suspected that it is sample data generated by different mechanisms.” Various application scenarios such as health detection systems, intrusion detection systems, bank risk assessment systems, and industrial fault monitoring systems will generate a large amount of high-dimensional complex data. Different from ordinary data mining, abnormal points or abnormal objects often carry more important and valuable information. For example, intrusion detection behavior in network security (Kwon et al., 2019), cash-out fraud in electronic digital payment systems (Abdallah et al., 2016), disease identification in medical field (Schlegl et al., 2019), and dangerous behavior detection in surveillance systems (Kiran et al., 2018). In the practical application of these systems, the occurrence of abnormal data means that there may be unfavorable situations, and it is necessary to conduct reasonable analysis and research on abnormal data in time to prevent and solve problems.

Over the past few years, many approaches have been developed in the field of anomaly detection. The most commonly used techniques include proximity-based and cluster-based techniques. The proximity measure of two data objects refers to a function of the proximity between the corresponding attributes of the two data objects. Distance is the most widely used and earliest proximity measure, so there are anomaly detection methods that define anomalies by the distance of the nearest neighbors. In addition to distance, another classic proximity measure is density, which was proposed by Breunig et al. (2000) to measure the degree of anomaly with the local outlier factor (LOF) for anomaly detection. Kernel density estimation (KDE) (Rosenblatt, 1958) is a common non-parametric method for detecting outliers. Latecki et al. (2007) proposed an outlier detection method using a kernel function. The outlier detection process is performed by comparing the local density of each point with the local density of its neighbors. In addition, most traditional anomaly detection techniques belong to the category of unsupervised learning, which mainly considers the distribution characteristics of the data. This is very similar to the working principle of the clustering algorithm, so the improved clustering algorithm can also be used as a means of anomaly detection. The main idea is to identify anomalies by comparing the differences between objects and clusters. Many related scholars have researched many classical clustering-based anomaly detection methods, such as DBSCAN (Ester et al., 1996), CLARANS (Ng and Han, 1994), CHAMELEON (George et al., 1999), BIRCH (Zhang et al., 1996), STING (Wang et al., 1997), WaveCluster (Sheikholeslami et al., 1998), CLIQUE (Agrawal et al., 1998), and FindCBOLF (He et al., 2003).

Due to the curse of dimensionality, many traditional anomaly detection methods are weak in modeling complex high-dimensional distributions. With the rapid development of deep learning, many scholars have developed methods to combine it with anomaly detection. The core idea of the one-class model coincides with the purpose of anomaly detection, that is, in the training phase, the model only learns the distribution characteristics of normal data. After the training is completed, the model identifies all data that does not belong to the normal class as abnormal data. Classic examples are one class SVM (Schölkopf et al., 1999) and support vector data description (SVDD) (Tax and Duin, 2004). With the development of subsequent research, deep anomaly detection models represented by autoencoder (AE) (Zhou and Paffenroth, 2017) and generative adversarial networks (GANs) have become mainstream methods (Kwon et al., 2019), but they all have their own limitations. AE-based anomaly detection models only focus on image reconstruction and cannot identify small anomalies, resulting in poor anomaly detection results. GAN-based anomaly detection models are generally difficult to train and difficult to converge.

In this paper, we propose a new method named anomaly detection with shared AEs based on the AE architecture (Zhou and Paffenroth, 2017), whose learning is inspired by GANs. Our core idea is to perform adversarial training on the proposed EDE structure, so that it learns to amplify the reconstruction error of abnormal data, and takes into account the reconstruction error of the latent vector. Compared with the method there, the performance is improved and stability is obtained. The main contributions of this paper are as follows:

• We propose an encoding-decoding-re-encoding network structure based on a shared AE, which takes into account both the reconstruction loss of the image and the reconstruction loss of the latent vector. This enables the model to find more anomalies.
• We also propose a two-stage training method that avoids the shortcomings of AEs and GANs. The second stage of adversarial training makes the training difficulty of the model lower, and it has better anomaly detection performance.

## 2. Related work

We emphatically introduce various anomaly detection methods based on two deep models.

### 2.1. Autoencoders approaches

Deep AEs play an important role in anomaly detection methods (Zhou and Paffenroth, 2017). Classic examples are AE, variational AE (VAE) (Kingma and Welling, 2013), and deep convolutional AE (DCAE) (Masci et al., 2011). Since these models only use normal samples for training, the reconstruction error of abnormal samples is much larger than that of normal samples, so as to achieve the purpose of abnormal detection. The abnormal samples can be detected by setting the reconstruction error as the abnormal score. In addition, there have been many recent advances in the research of anomaly detection combined with AEs. Fan et al. (2020) proposed a novel hybrid unsupervised method, which first integrated a convolutional AE and Gaussian process regression to extract features and remove anomalies from noisy data. Puzzle-AE (Salehi et al., 2020) combines the idea of Puzzle-solving with the AE so that the AE will learn more meaningful features during training to avoid overfitting. Shvetsova et al. (2021) proposed a new and powerful method for image anomaly detection. It relies on classical AE methods and a re-designed training pipeline to handle complex images, as well as robust methods for computing image anomaly scores.

### 2.2. GAN-based anomaly detection

Generative adversarial networks (GANs) (Goodfellow et al., 2014) is a deep learning model that produces fairly good outputs through the mutual game learning of two modules including the generative model and discriminative model in the framework. The combination of GAN and anomaly detection was first proposed by Schlegl et al. (2017). They train the GAN using normal images and then use the test set for validation after training. After inputting the test image into the GAN, by comparing the difference between the test image and the reconstructed image, it can be judged whether the image is normal or abnormal, and the abnormal area can also be judged. Akcay et al. (2019) proposed a network structure called GANomaly (Rosenblatt, 1958) to improve the above research. For abnormal data, the latent space vector obtained by the GANomaly structure is quite different from the latent space vector obtained after the first encoding, so that abnormal samples can be detected. One-class novelty detection using gans with constrained latent representations (OCGAN) (Perera et al., 2019) uses a denoising AE model to reconstruct the latent space vector and has better results after limiting the latent space vector. Perceptual image anomaly detection (PIAD) (Tuluptceva et al., 2019) proposes a new proximity metric that represents the perceptual proximity between images and is robust. In addition, this paper introduces a new method of choosing weights to make hyperparameter tuning more convenient. In AnoNAGN (Chen et al., 2020), a novel decoder-encoder framework is proposed to achieve a higher stability and lower training loss by employing a non-adversarial generative network.

## 3. Proposed method

### 3.1. Problem definition

We aim to train an unsupervised anomaly detection network that is trained using only normal samples while being able to distinguish between normal and abnormal samples when testing the network. The formal definition of this problem is as follows:

We give a large training dataset 𝒟 that only contains *M* normal images, 𝒟 = {*x*_1_, *x*_2_, …, *x*_*M*_}, and a small testing dataset $\hat{𝒟}$ containing M* both normal and abnormal images, $\hat{𝒟}=\left[\left({\hat{x}}_{1},y_{1}\right),\left({\hat{x}}_{2},y_{2}\right),\ldots ,\left({\hat{x}}_{M^{*}},y_{M^{*}}\right)\right]$, where *y*_*i*_ ∈ [0, 1] denotes the image of label. In specific training, the size of the training dataset needs to be much larger than the testing dataset such that *M* ≫ M*.

Given a dataset, we first need to model 𝒟 to learn its manifold, and then identify abnormal samples in $\hat{𝒟}$ as outliers during the inference phase. The model *f* learns the normal data distribution and minimizes the output anomaly score 𝒜(*x*). For any test image $\hat{x}$, if its abnormal score is too high, it may be judged as abnormal. The specific judgment criterion is based on the threshold *ϕ* of the abnormal score. If 𝒜(*x*) > *ϕ*, the image is judged as an abnormal image.

### 3.2. Network architecture

**FIGURE 1:**
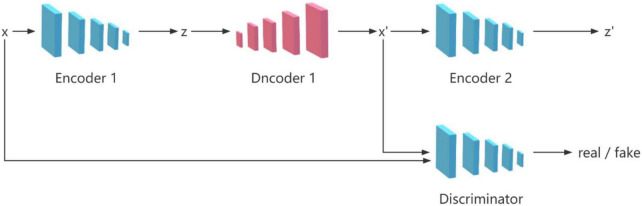
GANomaly architecture illustrating the information flow.

The GANomaly model (Rosenblatt, 1958) consists of four network structures, which are two encoders, one decoder and one discriminator, as shown in Figure 1. The model takes into account the differences between latent vectors and performs well in image anomaly detection, which makes many researchers improve on this model (Perera et al., 2019; Tuluptceva et al., 2019; Chen et al., 2020; Salehi et al., 2020; Shvetsova et al., 2021). The most important framework of the model is two encoders and one decoder, which are used to perform mutual transformation between the image and the latent vector, so as to identify abnormal data whose differences are amplified during the transformation process. The discriminator is used to distinguish the real image from the generated image.

**FIGURE 2:**
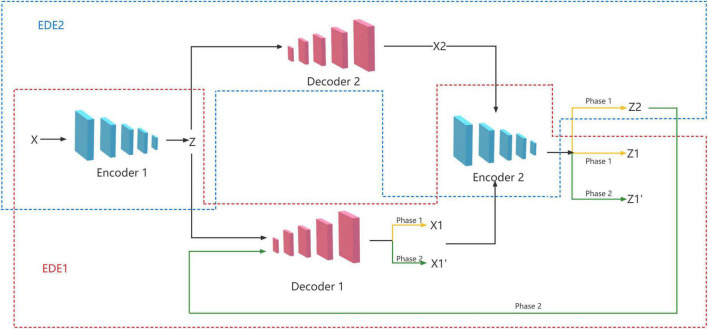
Proposed architecture illustrating the information flow.

In fact, the most important process in the GANomaly model is encoding, decoding, and re-encoding. Inspired by this, we propose an EDE structure, which only contains two encoders and one decoder. However, a single EDE structure needs to repeatedly adjust the relevant parameters to achieve better results, and it is difficult to meet the current needs of image anomaly detection. So we add an additional decoder to the EDE structure, and let the two decoders share the two encoders at the same time, and finally form the model of two EDE network structures, as shown in Figure 2.

**FIGURE 3:**
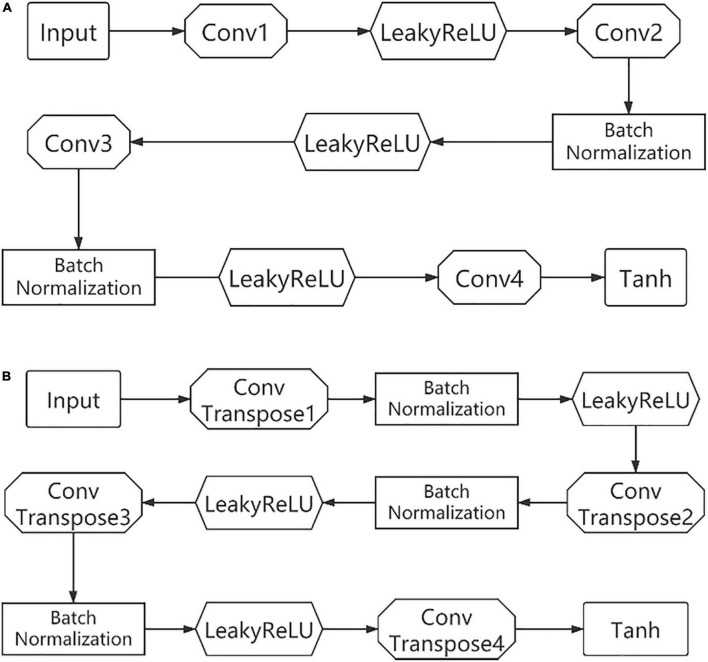
The specific structure of the encoder **(A)** and decoder **(B)**.

Two of the encoders use a similar structure, with four layers of convolutional layers. And the activation function uses LeakyReLU except for the Tanh used in the last layer. Some of the convolutional layers are followed by a batch normalization operation. The two decoders are similar in structure to the encoder except that each layer is replaced by ConvTranspose and an additional batch normalization, as shown in Figure 3.

### 3.3. Two-phase training

We propose a novel two-stage training method to achieve good performance with the network structure. In the first stage, we train two EDE structures to reconstruct images and latent vectors. In the second stage, we train these two EDE structures in an adversarial manner, where the purpose of EDE2 is to try to fool EDE1, and the purpose of EDE1 is to distinguish whether the data is real data or reconstructed data from EDE2.

#### 3.3.1 Phase 1: Reconstruction training

In the first stage, we need to train the EDE structure to reconstruct the input image and latent vector. First, the input image *x* is encoded by the encoder *E*_1_ and compressed into a latent vector *z*. The latent vector *z* is sent to the two decoders *D*_1_ and *D*_2_, respectively for decoding, and two reconstructed images *x*_1_ and *x*_2_ are obtained. Finally, we send the obtained two reconstructed images *x*_1_ and *x*_2_ to the encoder *E*_2_ for re-encoding, and get two reconstructed latent vectors *z*_1_ and *z*_2_. The training objectives of this stage are:


(1) $${ℒ}_{EDE_{1}}=\alpha {||x-x_{1}||}_{2}+\beta {||z-z_{1}||}_{2}$$

(2) $${ℒ}_{EDE2}=\alpha {||x-x_{2}||}_{2}+\beta {||z-z_{2}||}_{2}$$

Where *α*, *β* are the weighting parameters adjusting the impact of individual losses to the overall objective function.

#### 3.3.2 Phase 2: Adversarial training

In the second stage, we exploit the adversarial idea to train two EDE structures. The training goal of EDE1 is to distinguish between real data and data from EDE2. EDE2 is just the opposite and needs to deceive EDE1 as much as possible. First, the *z*_2_ obtained in the first stage is sent to the decoder *D*_1_ for decoding, and a new reconstructed image $x_{1}^{\prime }$ is obtained. Similarly, we send $x_{1}^{\prime }$ to the encoder *E*_2_ again to encode, and get a new latent vector $z_{1}^{\prime }$. The training objectives of this stage are:


(3) $$\underset{EDE_{2}}{\text{min}}\underset{EDE_{1}}{\text{max}}\alpha ||x-x_{1}^{\prime }||+\beta {||z-z_{1}^{\prime }||}_{2}$$

The respective loss functions of the two EDE structures are:


(4) $${ℒ}_{EDE_{1}}=-\alpha {||x-x_{1}^{\prime }||}_{2}-\beta {||z-z_{1}^{\prime }||}_{2}$$

(5) $${ℒ}_{EDE_{2}}=+\alpha {||x-x_{1}^{\prime }||}_{2}+\beta {||z-z_{1}^{\prime }||}_{2}$$

The parameters *α* and *β* are the same as those expressed above.

#### 3.3.3 Loss function

In our proposed structure, each EDE serves a dual purpose. EDE1 needs to minimize the reconstruction loss of *x* and *z* in phase 1. Meanwhile, EDE1 also needs to maximize the difference between *x* and $x_{1}^{\prime }$, and the difference between latent vectors *z* and $z_{1}^{\prime }$ in phase 2. The purpose of EDE2 in phase 1 is the same as that of EDE1, both to minimize the reconstruction error of *x* and *z*. But in phase 2, it is exactly the opposite of EDE1, which needs to minimize the difference between *x* and $x_{1}^{\prime }$, and the difference between *z* and $z_{1}^{\prime }$. The dual training objective for each EDE is a combination of the above loss functions, where the proportion of each part evolves over time:


(6) $$\begin{array}{l} {ℒ}_{EDE_{1}}=\frac{1}{n}\left(\alpha ||x-x_{1}\text{|}{\text{|}}_{2}+\beta ||z-z_{1}\text{|}{\text{|}}_{2}\right)-\left(1-\frac{1}{n}\right)\\ \left(\alpha ||x-x_{1}^{\prime \prime }\text{|}{\text{|}}_{2}+\beta ||z-z_{1}^{\prime \prime }\text{|}{\text{|}}_{2}\right) \end{array}$$

(7) $$\begin{array}{l} {ℒ}_{EDE_{2}}=\frac{1}{n}\left(\alpha ||x-x_{1}\text{|}{\text{|}}_{2}+\beta ||z-z_{1}\text{|}{\text{|}}_{2}\right)+\left(1-\frac{1}{n}\right)\\ \left(\alpha ||x-x_{1}^{\prime \prime }\text{|}{\text{|}}_{2}+\beta ||z-z_{1}^{\prime \prime }\text{|}{\text{|}}_{2}\right) \end{array}$$

Where *n* denotes a training epoch. The two-phase training process is summarized in Algorithm 1.

**Algorithm 1:** Model training algorithm.

**Input:** Normal images Dataset 𝒟 = {*x*_1_, *x*_2_, …, *x*_*M*_}, Number epochs *N*, Weighting parameters *α*, *β* **Output:** Trained EDE1, EDE2 *E*_1_, *E*_2_, *D*_1_, *D*_2_ ← initialize weights *n* ← 1 **repeat** **for** *m* = 1 to *M* **do** *z_m_* ← *E*_1_(*x*_*m*_) $x_{m}^{1}$ ← *D*_1_(*z*_*m*_) $x_{m}^{2}$ ← *D*_2_(*z*_*m*_) $z_{m}^{1}$ ← $E_{2}\left(x_{m}^{1}\right)$ $z_{m}^{2}$ ← $E_{2}\left(x_{m}^{2}\right)$ $x_{m}^{1}{}^{\prime }$ ← $D_{1}\left(z_{m}^{2}\right)$ $z_{m}^{1}{}^{\prime }$ ← E2(xm1)′ $\begin{array}{l} {ℒ}_{EDE_{1}}\leftarrow \frac{1}{n}\left(\alpha ||x_{m}-x_{m}^{1}\text{|}{\text{|}}_{2}+\beta ||z_{m}-z_{m}^{1}\text{|}{\text{|}}_{2}\right)\\ \;-\left(1-\frac{1}{n}\right)(\alpha ||x_{m}-x_{m}^{1}\prime \text{|}{\text{|}}_{2}+\\ \;\beta ||z_{m}-z_{m}^{1}\prime \text{|}{\text{|}}_{2} \end{array}$ $\begin{array}{l} {ℒ}_{EDE_{2}}\leftarrow \frac{1}{n}\left(\alpha ||x_{m}-x_{m}^{1}\text{|}{\text{|}}_{2}+\beta ||z_{m}-z_{m}^{1}\text{|}{\text{|}}_{2}\right)\\ \;+\left(1-\frac{1}{n}\right)(\alpha ||x_{m}-x_{m}^{1}\prime \text{|}{\text{|}}_{2}+\\ \;\beta ||z_{m}-z_{m}^{1}\prime \text{|}{\text{|}}_{2} \end{array}$ *E*_1_, *E*_2_, *D*_1_, *D*_2_ ← update weights using *ℒ*_*EDE*_1__ and *ℒ*_*EDE*_2__ **end for** *n* ← *n + 1* **until** *n* = *N*

#### 3.3.4 Anomaly score

During the detection phase, our model uses the following anomaly scores:


$$A(\hat{x})=\omega_{1}\text{||}z-z_{2}\text{|}{\text{|}}_{2}+\omega_{2}\text{||}z-z_{1}^{\prime }\text{|}{\text{|}}_{2}$$

Where *ω*_1_ + *ω*_2_ = 1 are weight parameters, which can adjust the ratio of true positives to false positives, thereby adjusting the sensitivity. In practical applications, we can adjust these two parameters to obtain anomaly detection results with different sensitivities in one experiment. The Algorithm 2 shows the testing process of the model.

**Algorithm 2:** Model testing algorithm.

**Input:** Testing Dataset $\hat{𝒟}=\left\{\left({\hat{x}}_{1},y_{1}\right),\left({\hat{x}}_{2},y_{2}\right),\ldots ,\left({\hat{x}}_{M^{*}},y_{M^{*}}\right)\right\}$, Threshold ϕ, Weighting parameters ω_1_, ω_2_ **Output:** Prediction label of testing dataset 𝒟_*pre*_ = {*y*_1*pre*_, *y*_2*pre*_, …, *y*_M*pre_} **for** *i* = 1 to M* **do** *z_i_* ← *E*_1_(*x*_*i*_) *x*_*2i*_ ← *D*_2_(*z*_*i*_) *z*_*2i*_ ← *E*_2_(*x*_2*i*_) $z_{1i}^{\prime }$ ← *D*_1_(*z*_2*i*_) $z_{1i}^{\prime }$ ← $E_{2}\left(z_{1i}^{\prime }\right)$ $A\left({\hat{x}}_{i}\right)\leftarrow \omega_{1}{||z_{i}-z_{2i}||}_{2}+\omega_{2}{||z_{i}-z_{1i}^{\prime }||}_{2}$ **if** $A\left({\hat{x}}_{i}\right)\geq \phi$ **then** *y*_*ipre*_ ← 1 **else** *y*_*ipre*_ ← 0 **end if** **end for**

## 4. Experiments

### 4.1. Datasets

**FIGURE 4:**
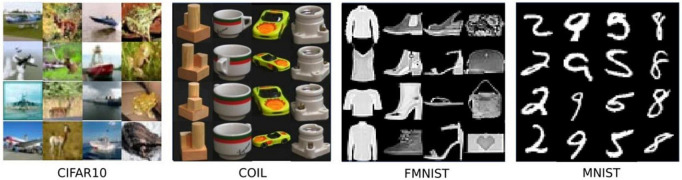
Representative images from four datasets. Each column presents the same class.

In this section, we briefly introduce each dataset used for evaluation. In Figure 4, some representative examples from the considered dataset are shown.

#### 4.1.1 COIL-100

The COIL-100 dataset is a dataset composed of different objects imaged at different angles in a 360° rotation. It contains 128 × 128 color images of 100 objects (each with 72 poses), and 49,152 features (red, green, and 128 × 128 pixel value in the blue channel).

#### 4.1.2 MNIST

The MNIST dataset is very classic in the field of machine learning. It consists of 60,000 training samples and 10,000 test samples, each of which is a 28 × 28 pixel grayscale handwritten digit picture. The complexity of the MNIST dataset is more challenging than COIL-100.

#### 4.1.3 fMNIST

fMNIST is an image dataset that replaces the MNIST handwritten digits set. It is the same size and format as the original MNIST, with 70,000 grayscale images of fashion products, and the image size is 28 × 28.

#### 4.1.4 CIFAR-10

It is a set of color image data and is a commonly used public benchmark dataset in the fields of machine learning and computer vision. It contains 10 different categories of images, each category contains 6,000 32 × 32 × 3 color images with a total of 784 pixels. Among the many datasets for anomaly detection, CIFAR-10 is the most challenging dataset due to its content diversity and complexity.

#### 4.1.5 CIFAR-100

This dataset is similar to CIFAR-10 and consists of 60,000 32 × 32 × 3 color images. The 100 classes in the CIFAR-100 are grouped into 20 superclasses. There are 600 images per class. Each image comes with a “fine” label (the class to which it belongs) and a “coarse” label (the superclass to which it belongs). There are 500 training images and 100 testing images per class.

### 4.2. Experimental setup and evaluation methodology

To test the proposed method, we use a well-accepted anomaly detection method (Schlegl et al., 2017; Perera et al., 2019; Tuluptceva et al., 2019; Chen et al., 2020): we use a one-vs.-all protocol to treat a certain class in a multi-class dataset as a normal class, then treat all other classes as anomalous classes, and finally traverse all the classes in a loop. In the training process, we only use the normal class data to train the model. In the testing process, we mix the normal class data with the abnormal class data as the test set. At the same time, we also noticed that there are two methods of dividing the training set and the test set in the literature.

(1) Use 80% of the normal class data for training and the remaining 20% of the normal class data for testing. The abnormal class test data are randomly selected, and their number is the same as the 20% normal class data.
(2) Experiment using the training and test sets given by the dataset. The training split of the normal class is used for training and validation. Test data for all classes is used for testing.

In our experiments, we adopted two partitioning methods for different datasets according to the methods of mainstream literature.

### 4.3. Experimental results

We first picked several classical methods as standards, such as VAE (Kingma and Welling, 2013) and one class-support vector machine (OC-SVM) (Schölkopf et al., 1999). Meanwhile, a variety of state-of-the-art method about anomaly detection are selected for comparison with our proposed method, including AnoGAN (Schlegl et al., 2017), pixel CNN (Van den Oord et al., 2016), Deep-SVDD (Ruff et al., 2018), OCGAN (Perera et al., 2019), PIAD (Tuluptceva et al., 2019), robust deep auto-encoding gaussian process regression for unsupervised anomaly detection (DAGPR) (Fan et al., 2020), generative probabilistic novelty detection with adversarial autoencoders (GPND) (Pidhorskyi et al., 2018), Puzzle-AE (Salehi et al., 2020), AnoNAGN (Chen et al., 2020), and anomaly detection in medical imaging with deep perceptual autoencoders (ADDPA) (Shvetsova et al., 2021).

We use the first method of splitting the training and testing sets in section “4.2 Experimental setup and evaluation methodology” to conduct experiments on the MNIST, f-MNIST, and COIL-100 datasets (Perera et al., 2019; Tuluptceva et al., 2019; Chen et al., 2020). For the COIL-100 dataset, we randomly select one class as the normal class and the other classes as the abnormal class for anomaly detection, and then repeat this process 30 times (Pidhorskyi et al., 2018). For the f-MNIST and MNIST dataset, we loop each class as a normal class for anomaly detection. Table 1 shows the comparison results of our method with other methods. Our method outperforms the state-of-the-art methods by 0.7 and 0.2% on the MNIST and f-MNIST datasets, and achieves an area under curve (AUC) of 1 on the COIL-100 dataset.

**TABLE 1:** Average receiver operating characteristic curve (ROC) area under curve (AUC) for anomaly detection on fMNIST and COIL-100.

Model	MNIST	fMNIST	COIL-100
GPND	0.932	0.933	0.979
OCGAN	0.977	0.924	0.995
PIAD	0.979	0.949	**1.000**
AnoNAGN	0.985	0.995	**1.000**
Proposed method	**0.992**	**0.997**	**1.000**

Bold values indicate that this value is the highest.

For the MNIST and CIFAR-10 datasets, we use the second method in section “4.2 Experimental setup and evaluation methodology” to split the training set and the dataset. We conduct three experiments for each class of the dataset and select the average AUC values shown in Tables 2, 3.

**TABLE 2:** Anomaly detection results for MNIST dataset.

Model	0	1	2	3	4	5	6	7	8	9	Mean
VAE	0.997	**0.999**	0.936	0.959	0.973	0.964	0.993	0.976	0.923	0.976	0.970
OCSVM	0.988	**0.999**	0.902	0.950	0.955	0.968	0.978	0.965	0.853	0.955	0.951
AnoGAN	0.966	0.992	0.850	0.887	0.894	0.883	0.947	0.935	0.849	0.924	0.913
PixelCNN	0.531	0.995	0.476	0.517	0.739	0.542	0.592	0.789	0.340	0.662	0.618
Deep-SVDD	0.980	0.997	0.917	0.919	0.949	0.885	0.983	0.946	0.939	0.965	0.948
OCGAN	**0.998**	**0.999**	0.948	0.963	0.975	0.980	0.991	0.981	0.939	0.981	0.975
PIAD	0.996	**0.999**	0.985	0.981	0.960	0.976	0.995	0.984	**0.982**	0.989	0.985
DAGPR	0.993	0.993	0.979	0.959	0.949	0.934	0.970	0.951	0.943	0.938	0.961
Puzzle-AE	0.996	**0.999**	0.971	0.970	**0.977**	0.984	0.992	0.983	0.941	0.986	0.980
AnoNAGN	**0.998**	**0.999**	0.987	**0.986**	0.965	**0.989**	**0.998**	0.992	0.970	0.979	0.983
ADDPA	0.997	**0.999**	0.986	0.984	0.964	0.983	0.996	0.989	0.976	0.981	0.986
Ours	0.986	**0.999**	**0.999**	**0.999**	**0.988**	**0.999**	**0.998**	**0.992**	0.979	**0.999**	**0.994**

Bold values indicate that this value is the highest.

**TABLE 3:** Anomaly detection results for CIFAR-10 dataset.

Model	Plane	Car	Bird	Cat	Deer	Dog	Frog	Horse	Ship	Truck	Mean
VAE	0.700	0.386	0.679	0.535	0.748	0.523	0.687	0.493	0.696	0.386	0.583
OCSVM	0.630	0.440	0.649	0.487	0.735	0.500	0.725	0.533	0.649	0.508	0.586
AnoGAN	0.671	0.547	0.529	0.545	0.651	0.603	0.585	0.625	0.758	0.665	0.618
PixelCNN	0.788	0.428	0.617	0.574	0.511	0.571	0.422	0.454	0.715	0.426	0.551
Deep-SVDD	0.617	0.659	0.508	0.591	0.609	0.657	0.677	0.673	0.759	0.731	0.648
OCGAN	0.757	0.531	0.640	0.620	0.723	0.620	0.723	0.575	0.820	0.554	0.657
PIAD	0.837	0.876	0.753	0.602	0.808	0.713	0.839	0.842	0.867	0.849	0.799
DAGPR	0.751	0.737	0.595	0.564	0.692	0.572	0.692	0.531	0.767	0.793	0.669
Puzzle-AE	0.789	0.780	0.700	0.549	0.755	0.660	0.748	0.733	0.833	0.700	0.725
AnoNAGN	**0.962**	0.638	0.725	0.643	0.873	0.638	0.883	0.584	0.935	0.645	0.750
ADDPA	0.865	**0.922**	**0.768**	0.587	0.851	**0.777**	0.889	**0.891**	0.914	**0.922**	0.839
Ours	0.925	0.867	0.757	**0.894**	**0.940**	0.754	**0.963**	0.818	**0.917**	0.834	**0.867**

Bold values indicate that this value is the highest.

Since the pictures in the MNIST dataset are single-channel grayscale images and the data dimension is not high, most models can achieve good results. But our model leads in 8 out of 10 categories and achieves a performance lead of 0.8% on average AUC.

**FIGURE 5:**
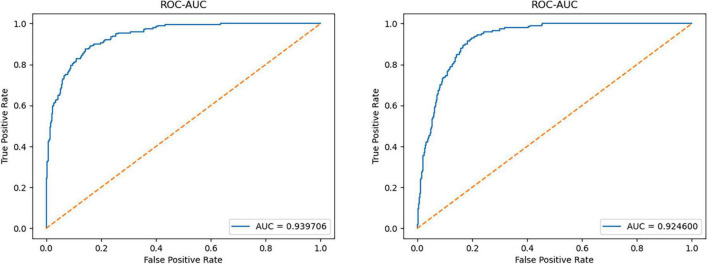
The ROC curve of the model in the plane and deer categories in CIFAR-10.

For the CIFAR-10 dataset, the diversity and complexity of images is greatly improved, which makes most models perform poorly. However, our method still achieves a performance advantage compared with the most novel methods, which is largely due to the model taking into account the reconstruction loss of the latent space vectors. Among them, when some categories (such as cat, deer, and frog) are used as normal categories, the performance improvement of our model is obvious. The average AUC value also achieved a 2.8% lead. Figure 5 shows the ROC curves of the model when detecting two types of anomalies.

In addition, in order to prove the effectiveness of the proposed model for anomaly detection tasks in complex images, we also conducted experimental verification on CIFAR-100. We randomly select 10 of the 20 superclasses in CIFAR-100, and randomly select one subclass from these 10 superclasses as normal class, and the other four subclasses as abnormal class for experiment. Since the subclasses within a superclass all have varying degrees of similarity, this is a great challenge for most anomaly detection models. Table 4 shows the AUC values and the average AUC values of various models tested in the CIFAR-100 dataset. The performance of most models in this data set has declined, but our model is more robust and still takes the lead in five categories, including difficult categories (such as clock and bee), and the average AUC value achieved a 3.4% lead.

**TABLE 4:** Anomaly detection results for CIFAR-100 dataset.

Model	Shark	Cups	Apples	Clock	Bed	Bee	Bear	Cloud	Fox	Bus	Mean
VAE	0.588	0.432	0.658	0.512	0.676	0.491	0.614	0.523	0.621	0.439	0.555
OCSVM	0.612	0.429	0.615	0.533	0.682	0.478	0.716	0.548	0.579	0.469	0.566
AnoGAN	0.658	0.531	0.563	0.537	0.734	0.583	0.569	0.601	0.648	0.647	0.607
PixelCNN	0.754	0.431	0.695	0.558	0.549	0.553	0.437	0.449	0.603	0.527	0.556
Deep-SVDD	0.748	0.637	0.656	0.584	0.593	0.634	0.537	0.681	0.723	0.606	0.640
OCGAN	0.739	0.581	0.632	0.638	0.697	0.614	0.686	0.601	0.808	0.599	0.660
PIAD	0.829	0.859	0.768	0.614	0.789	0.687	0.817	0.851	0.831	0.865	0.791
DAGPR	0.748	0.729	0.689	0.609	0.731	0.593	0.683	0.615	0.763	0.751	0.691
Puzzle-AE	0.801	0.769	0.724	0.637	0.749	0.681	0.782	0.701	0.812	0.685	0.734
AnoNAGN	0.833	0.713	**0.904**	0.756	0.848	0.718	0.853	0.776	0.953	0.724	0.808
ADDPA	0.841	**0.893**	0.714	0.761	0.855	0.711	**0.914**	**0.823**	0.924	**0.917**	0.835
Ours	**0.931**	0.872	0.809	**0.857**	**0.938**	**0.739**	0.897	0.819	**0.961**	0.871	**0.869**

Bold values indicate that this value is the highest.

To investigate the effectiveness of each additional component of the proposed work, we performed an ablation study using the MNIST dataset. Specifically, we consider four cases. In the first case, we only consider AEs. In the second case, we use a set of EDE structures without adversarial training. In the third case, we use two sets of EDE structures without adversarial training. In the last scenario, we use the proposed full model. The average AUCs for each class of MNIST datasets are listed in Table 5.

**TABLE 5:** Ablation study for proposed model performed on MNIST.

One EDE	0.961
Two EDE without adversarial training	0.974
Two EDE + Adversarial training	0.989

## 5. Conclusion

We introduce a deep anomaly detection method by building a model with two EDE structures and using a two-stage training method. For the former, the emergence of the EDE model makes the training goal not only focus on the reconstruction of the image, but also reduce the loss of the latent vector. The two-stage method uses the idea of adversarial training to avoid the shortcomings of AEs and GANs.

We demonstrate our method outperforms other state-of-the-art deep learning methods on multiple image datasets above. For future work, we can generalize the model to high-resolution images and more general domains such as medical imaging or security imaging. We also want to explore the applicability of the model to anomaly detection in videos. In addition, other generative tasks are also optional research directions, such as image reconstruction.

## Data availability statement

The original contributions presented in this study are included in the article/supplementary material, further inquiries can be directed to the corresponding author.

## Author contributions

HJ: conceptualization, methodology, software, and writing—original draft preparation. WL: writing—review and editing and supervision. Both authors contributed to the article and approved the submitted version.
